# *Toxoplasma gondii* serine-protease inhibitor-1: A new adjuvant candidate for asthma therapy

**DOI:** 10.1371/journal.pone.0187002

**Published:** 2017-10-26

**Authors:** Ariadna S. Soto, Ignacio M. Fenoy, Vanesa R. Sanchez, Florencia March, Matías D. Perrone Sibilia, María de los Angeles Aldirico, Mariano S. Picchio, Nadia Arcon, Patricio L. Acosta, Fernando P. Polack, Valentina Martin, Alejandra Goldman

**Affiliations:** 1 Laboratorio de Inmunología, Vacunas y Alergia, CESyMA, Escuela de Ciencia y Tecnología, Universidad Nacional de San Martín, Buenos Aires, Argentina; 2 Consejo Nacional de Investigaciones Científicas y Técnicas (CONICET), Buenos Aires, Argentina; 3 Fundación Infant, Buenos Aires, Argentina; Mie Daigaku, JAPAN

## Abstract

Serine-proteases are important players in the pathogenesis of asthma, promoting inflammation and tissue remodeling. It’s also known that many serine protease inhibitors display immunomodulatory properties. TgPI-1 is a *Toxoplasma gondii* protein that exhibits broad spectrum inhibitory activity against serine proteases. In view of the increased prevalence of atopic disorders and the need to develop new treatment strategies we sought to investigate the potential of TgPI-1 for treating respiratory allergies. For this purpose, we developed a therapeutic experimental model. BALB/c mice were rendered allergic by intraperitoneal ovalbumin-alum sensitization and airway-challenged. Once the asthmatic phenotype was achieved, mice were intranasally treated with rTgPI-1 alone or with a mixture of rTgPI-1 and ovalbumin (OVA). A week later mice were given a secondary aerosol challenge. Treatment with rTgPI-1 alone or co-administered with OVA diminished bronchoalveolar eosinophilia, mucus production and peribronchial lung infiltration. This effect was accompanied by a lung resistance reduction of 26.3% and 50.3% respectively. Both treatments resulted in the production of lower levels of IL-4, IL-5, IFN-γ and regulatory IL-10 by thoracic lymph node cells stimulated with OVA. Interestingly, significant decreases in OVA specific IgE and T cell proliferation, and increases in FoxP3^+^ T cells at local and systemic levels were only detected when the inhibitor was administered along with OVA. These results show that both rTgPI-1 treatments reduced asthma hallmarks. However, co-administration of the inhibitor with the allergen was more effective. Hence, rTgPI-1 emerges as a novel adjuvant candidate for asthma treatment.

## Introduction

Asthma is a chronic inflammatory disease of the airways characterized by infiltration of the pulmonary mucosa with eosinophils, macrophages and T cells, goblet cell metaplasia and epithelial hypertrophy/hyperplasia. This inflammatory condition orchestrated by Th2-type cytokines leads to airway obstruction with an exacerbated response to different stimuli (AHR), and bronchial architecture remodeling with subepithelial fibrosis [1]. Present therapies for treatment of allergic conditions depend on antihistamines and anti-inflammatory agents; however, the long term use of these drugs may lead to the appearance of side effects and the generation of resistance [2–4]. Therefore, immunotherapy is currently an alternative treatment to antihistamines, bronchodilators and corticosteroids.

It is well known that allergy may be down modulated by certain infections [5]. The knowledge of the mechanisms participating in this process offered novel therapeutic approaches for allergic diseases by using immunomodulatory microbe’s components [6,7]. In this context, we [8] and others [9] have previously shown that *Toxoplasma gondii* infection decreases the subsequent development of allergic lung inflammation. These results led us to search for parasite proteins with immunomodulatory properties that could be used for allergy treatments. The *T*. *gondii* protease inhibitor-1 (TgPI-1) is a four domain protein that belongs to Kazal-type serine protease inhibitors [10,11]. Biochemical studies with a recombinant form of this protein have shown its ability to inhibit a broad range of serine-proteases, including trypsin, chymotrypsin, neutrophil elastase [12] and subtilisin [13]. It has been reported that many serine protease inhibitors such as the secretory leukocyte protease inhibitor (SLPI) and the serum protease inhibitor α1-antitrypsin (hAAT) possess immunomodulatory properties. These properties include the inhibition of inflammatory responses by preventing the activation of nuclear factor κB (NF-κB) [14] or by up-regulating IL-10 and TGF-β production by macrophages [15]; the development of antigen-specific T regulatory cells [16,17] and the reduction of B-lymphocyte responses [18].

In addition, it is known that both endogenous and exogenous proteases play significant roles in asthma pathophysiology [19–21]. Mast cell serine proteases [22], matrix metalloproteinase-9 (MMP-9) and neutrophil elastase [23] showed to be present in elevated levels in the airways of asthmatic patients suggesting an imbalance in the protease/anti-protease system. In this regard, it has been demonstrated that neutrophil elastase promotes bronchoconstriction [24] and stimulates the secretion of mucin5AC from airway epithelial cells [25]. Also, it has been shown that trypsin can activate human eosinophils [26].

Therefore, targeting proteolytic activity with inhibitors could lead to a reduction of proteases induced inflammatory diseases. Moreover, the immunomodulating ability of serine proteases inhibitors makes these proteins interesting to be studied as candidates for asthma immunotherapy. In this regard, and so far, the effect of serine protease inhibitors on allergic inflammation have mostly been studied in monotherapy protocols by treating the animals without allergen [27–30].

With this background and facing the need to find new molecules for the treatment of allergies, we analyzed the potential of the serine protease inhibitor-1 from *T*. *gondii* for treating respiratory allergies. For this purpose, we studied its efficacy either administered alone or in combination with the allergen. We found that both treatments with rTgPI-1 ameliorate the disease manifestations. Moreover, administration of the serine-protease inhibitor concomitantly with the allergen resulted in decreased allergen-specific T cell proliferation and expansion of FoxP3^+^ Tregs.

## Materials and methods

### Animals

BALB/c (H-2^d^) mice were obtained from the animal facilities of the School of Veterinary Sciences, University of Buenos Aires, Argentina, and maintained in our animal facilities for use throughout these experiments. Mice were used at the age of 6 to 8 weeks. All procedures requiring animals were approved by the Independent Ethics Committee for the Care and Use of Experimental Animals of National University of San Martin (CICUAE protocol ID # 005/16).

### Expression and purification of the recombinant protein

The recombinant TgPI-1 protein (rTgPI-1) was previously generated by Pszenny et al. [10]. The rTgPI-1 coding region of the mature protein (from residue 25 to the end, residue 294) was cloned into the BamHI and KpnI restriction sites of the pQE30 expression vector, which produces a recombinant protein with a 6x-His tag at the N-terminus (rTgPI-1). The recombinant protein includes the four kazal-type domains. *Escherichia coli* M15 transformed with plasmid pQE30-TgPI-1 was kindly provided by Dr. Angel (IIB-INTECH, UNSAM). For expression, as previously described [10,31,32], a single colony was grown overnight at 37°C in Luria-Bertani broth supplemented with ampicillin (100 μg/ml) and kanamycin (50 μg/ml), diluted ten times and grown at 37°C until OD600nm = 0.4. rTgPI-1 expression was induced by the addition of isopropyl-β-d-thiogalactopyranoside at final concentration of 1 mM. After 3 h of growth, cells were pelleted and resuspended in lysis buffer (8M Urea; 20 mM NaH_2_PO_4_; 300 mM NaCl; 10 mM imidazole; pH 7.4–7.6). Cells were lysed on ice by sonication and centrifuged at 10,000×g at 4°C for 20 min to prepare a supernatant. The supernatant was loaded onto the Ni^+2^-nitriloacetic acid resin affinity column (GE Healthcare Life Sciences), previously equilibrated with lysis buffer. The column was then washed with 10 volumes of wash buffer (20 mM NaH_2_PO_4_; 0.5 M NaCl; 20 mM imidazole; pH 7.4) and eluted with elution buffer (20 mM NaH_2_PO_4_; 0.5M NaCl; 250 mM imidazole; pH 7.4). The purified recombinant protein was quantitated by Bradford’s method.

Before mouse treatment, rTgPI-1 was dialyzed against PBS and endotoxins were removed with Detoxi-Gel™ Endotoxin Removing Columns (Thermo Fisher Scientific, Pierce Biotechnology), filtered throughout a 0.22 μm-pore membrane and stored at −80°C.

### Western blot analysis

For Western blot analysis, purified protein was incubated at 100°C for 5 min in loading buffer, separated by SDS-PAGE (12% gel) and transferred onto PVDF membrane (GE) using an Electro-transfer unit (Bio-Rad). The membranes were sequentially incubated with mouse polyclonal anti-rTgPI-1 antibody (1:1000) or anti-Histidine (1:3000; Sigma) and alkaline phosphatase conjugated goat anti-mouse IgG (1:5000; Sigma). After washing, the reaction was developed by the addition of nitroblue tetrazolium/5-bromo-4-chloro-3-indolyl phosphate (NBT/BCIP) substrate. PageRuler^TM^ Prestained Protein Ladder (Thermo Scientific) was used as molecular marker.

### Trypsin-inhibitory activity

Trypsin-inhibitory activity of rTgPI-1 was checked before mouse treatment by 12.5% SDS-PAGE in 0.15% gelatin containing gels, without prior reduction and boiling, a technique commonly used for detection of proteolytic activity [10]. Porcine trypsin (50 ng) (Sigma-Aldrich) was incubated with or without 1 μg of rTgPI-1 in the activity buffer (25 mM Tris HCl, pH 7.2, 50 mM NaCl) at 37°C for 15 min and then subjected to electrophoresis. The gel was washed twice with 0.1% Triton X-100, 20 min each, incubated at 37°C in the activity buffer overnight and stained with Coomassie Brilliant Blue R-250. The gelatinase activity was visualized as white bands on a blue background, which represented areas of proteolysis.

### Experimental protocol

**Fig 1:**
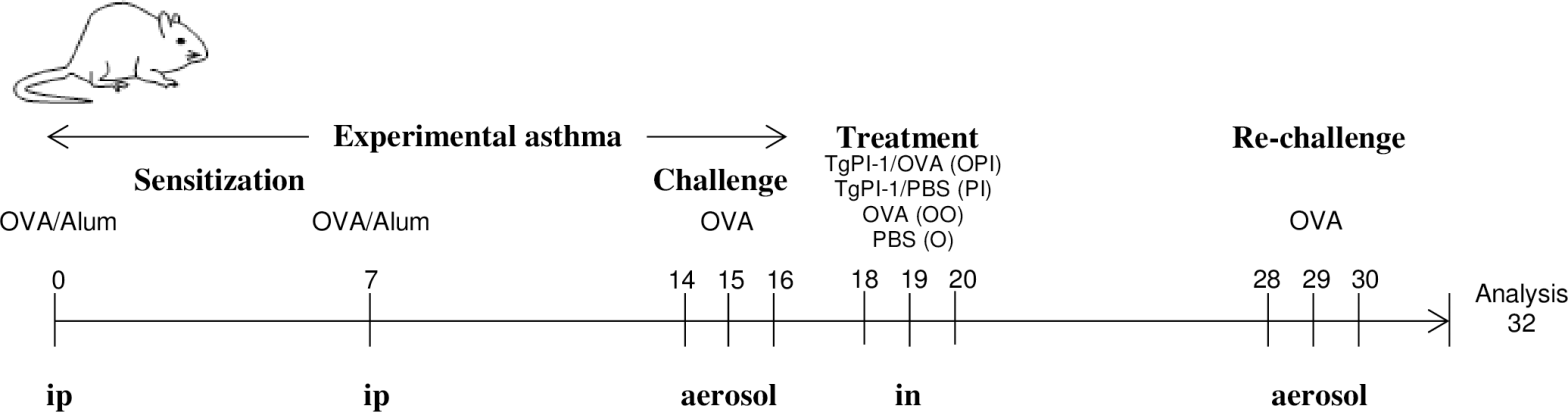
Schematic of asthma model and treatment protocol. Mice were sensitized by two injections of OVA/alum and challenged by exposure to aerosols of allergen on 3 consecutive days. Two days later mice were intranasally (in) treated with rTgPI-1 (PI) or rTgPI-1 plus OVA (OPI). Controls included non-sensitized mice treated with rTgPI-1 alone (N), and sensitized mice exposed intranasally to PBS (O) or OVA (OO). One week later, mice were re-challenged and analysis was performed 48 h after the last exposure.

Sensitization was achieved by two intraperitoneal injections of 0.2 ml PBS containing chicken egg white albumin (OVA) (grade V Sigma-Aldrich) (20 μg) and alum (2 mg) one week apart. One week later, mice were exposed to aerosols of allergen (3% (w/v)) OVA in PBS for 20 min on 3 consecutive days. Aerosol exposure was performed within individual compartments of a mouse pie chamber using a nebulizer (SAN-UP, Argentina, OVA solution flux 0.33 ml/min in air flux of 6–8 l/min). Forty eight hours after the last exposure, mice were intranasally treated with 50 μg of rTgPI-1 (PI) or a mixture of rTgPI-1 (50 μg) plus OVA (20 μg) (OPI) in a final volume of 35 μl. Controls included non-sensitized mice treated with rTgPI-1 alone (N), and sensitized mice exposed intranasally to PBS (O) (positive control of PI group) or OVA (OO) (positive control of OPI group). One week later, mice were re-challenged by exposing them to aerosols of OVA during 3 consecutive days. Analysis was performed 48 h after the last exposure. The experimental protocol is summarized in Fig 1.

### Pathologic analysis

Animals were euthanized with isoflurane. The chest wall was opened and the animals were exsanguinated by cardiac puncture. Serum was prepared and stored at -20°C. The trachea was cannulated after blood collection. Bronchoalveolar lavage (BAL) was performed four times with 1 ml of sterile PBS. Lavage fluid was collected, centrifuged at 300xg for 10 min, and the pellet was resuspended in 0.5 ml PBS. BAL differential cell counts were performed on cytocentrifuge slides prepared by centrifugation of samples at 300xg for 5 min (TD4-WS, Pingfan Instrument And Meter Co, Ltd.). These slides were fixed and stained with a modified Wright-Giemsa stain (Tinción 15, Biopur SRL, Rosario, Argentina), and a total of 200 cells were counted for each sample by optical microscopy. For histopathology, lungs were removed and fixed with formalin. Following paraffin embedding, tissue sections for microscopy were stained with H&E and Periodic acid-Schiff (PAS). An index of pathologic changes in H&E slides was obtained by scoring the inflammatory infiltrates around the airways and vessels for greatest severity (0, normal; 1, ≤ 3 cells diameter thick; 2, 4–10 cells diameter thick; 3, > 10 cells diameter thick) and overall extent (0, normal, 0.5, <5% of sample, 1, <25%, 2, 25–50%, 3, 51–75%, 4, >75%). The index was calculated by multiplying severity by extent. An histological goblet cell score was obtained in PAS-stained lung sections by examining 20 consecutive airways from all groups of mice at 400x magnification and categorized according to the abundance of PAS-positive goblet (0, <5% goblet cells; 1, 5–25%; 2, 26–50%; 3, 51–75%; 4, >75%). The index was calculated by dividing the sum of the airway scores from each lung by the number of airways examined for the histological goblet cell score [33].

### Lung function

Twenty four hours after the last challenge mice were anesthetized by an intraperitoneal injection of ketamine/xylazine. Six mice per group were tracheostomized and intubated to a small animal ventilator (FinePointe Series RC Sites, Buxco Research System, Wilmington, DE, USA). Then, the dynamic airway pressure was measured for 3 min and the maximal resistance measurement was obtained both before and after increased doses of aerosolized methacholine (0.0–30 mg/mL).

### *Ex vivo* cytokine production and proliferation assays

Single cell suspensions from thoracic lymph nodes (TLN) and spleen were made using a cell strainer and 3x10^5^ cells were cultured in 200 μl of medium RPMI 1640 supplemented with 20% FBS (NATOCOR, Córdoba, Argentina), 1% antibiotics and 5x10^-5^ M 2-mercaptoethanol alone or in the presence of OVA (grade V; Sigma-Aldrich) (200 μg/ml for TLN or 500 μg/ml for splenocyte proliferation). Cytokine production was measured in supernatants at 72 h by capture ELISA commercial kits (BD Biosciences). Proliferative responses were determined after addition of methyl-^3^[H]-thymidine (1 μCi/well, PerkinElmer, Argentina) for the last 18 h of a 5 days culture period. Proliferation is shown as the difference in incorporation of [^3^H]-thymidine between stimulated and non-stimulated cells (Δ cpm).

### Assay of serum immunoglobulin

ELISA plates (Nunc Maxisorp, Boston, MA, USA) were coated with OVA (10 μg/ml) in carbonate buffer (pH = 9.5) and placed at 4°C overnight. Mouse sera were diluted 1:40 (IgE), 1:64x10^6^ (IgG1) and 1:16x10^3^ (IgG2a). Biotinylated anti-IgE mouse antibody (BD, Biosciences) or HRP-conjugated goat anti-mouse IgG1 or IgG2b (BD, Biosciences) were used as a secondary antibodies. For IgE determination, streptavidin coupled to peroxidase enzyme (HRP, horseradish peroxidase-streptavidin, Zymed, 1/4000) was added. Immune complexes were revealed with trimethylbenzidine substrate (TMB One-Step; Dako, Carpenteria, CA, USA). Plates were read in a plate reader (Sunrise RC, Tecan) at 450 nm with λ correction at 570 nm after the addition of stop solution (H_2_SO_4_). Results are shown as optical density (OD) for a fixed dilution.

### Flow cytometry analysis

Intracellular staining of FoxP3 was performed using phycoerythrin-conjugated anti-FoxP3 mAb and the FoxP3 staining buffer set (Pharmingen, BD Biosciences) according to the manufacturer’s protocol. PerCP-Cy5.5-conjugated anti-CD4 mAb (clone RM4-5) from Pharmingen, BD was used. Cells were acquired on a FACScan cytometer (Becton Dickinson, Mountain View, CA). Data were analyzed by using FlowJo software (Tree Star, CA, USA)

### Statistical analysis

Each experimental group had at least four mice and each experiment was repeated at least 3 times. Data are presented as mean ± SEM. Statistical analysis was performed using ANOVA analysis of differences among groups with Bonferroni’s test “*a posteriori*” as indicated in the figure legends. Statistical significance was accepted when p<0.05.

## Results

### Purification and activity of rTgPI-1

**Fig 2:**
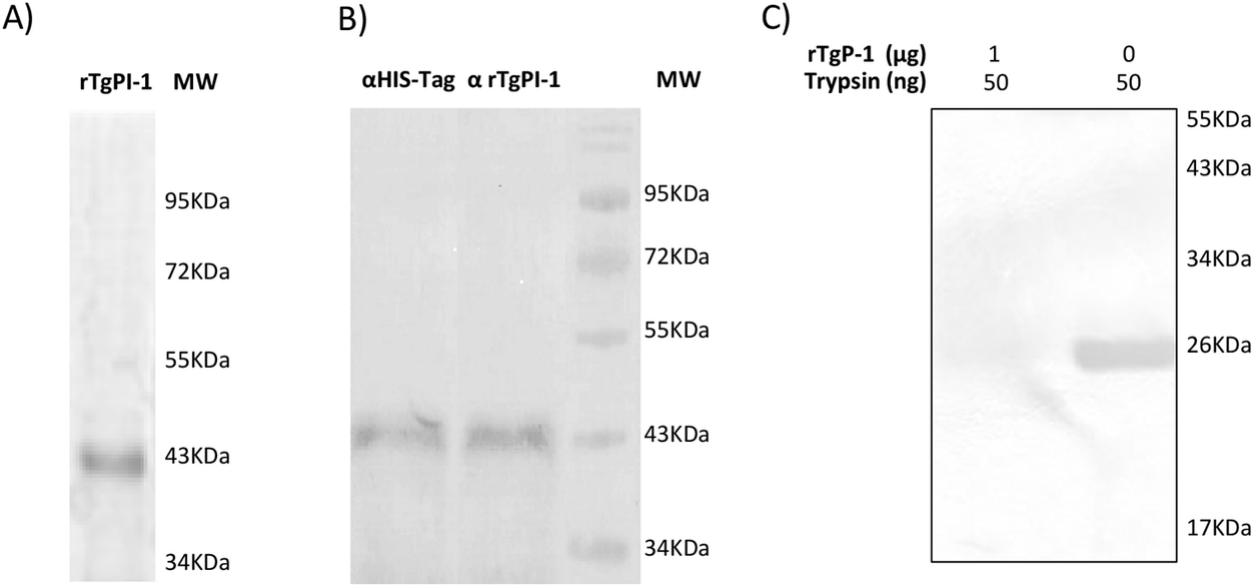
Purification and activity of rTgPI-1. (A) SDS-PAGE of rTgPI-1 stained with Coomassie Blue. (B) Western blot of rTgPI-1 revealed with anti-His-Tag (lane 1) antibody or mouse anti-TgPI-1 serum (lane 2). (C) Analysis of inhibitory effect of rTgPI-1 on trypsin by gelatin substrate-SDS PAGE. All lanes contain 50 ng of trypsin. Lane 1, 1 μg of rTgPI-1; lane 2, control without rTgPI-1. Gelatinolytic activity was visualized by staining with Coomassie Brilliant Blue. The image was digitally inverted.

The purified rTgPI-1 protein migrated as a unique band with an apparent molecular weight of ∼42 kDa (Fig 2A), which was recognized by a mouse polyclonal antibody against rTgPI-1 (Fig 2B). To confirm the activity of the purified rTgPI-1, the ability to inhibit trypsin was analyzed by SDS-PAGE zymography. Fig 2C shows that trypsin generated a band indicating protease activity (lane 2). After incubation in the presence of rTgPI-1 the gelatinolytic activity was completely abolished (lane 1).

### Intranasal Administration of rTgPI-1 Suppresses Allergen-Induced Airway Inflammation

**Fig 3:**
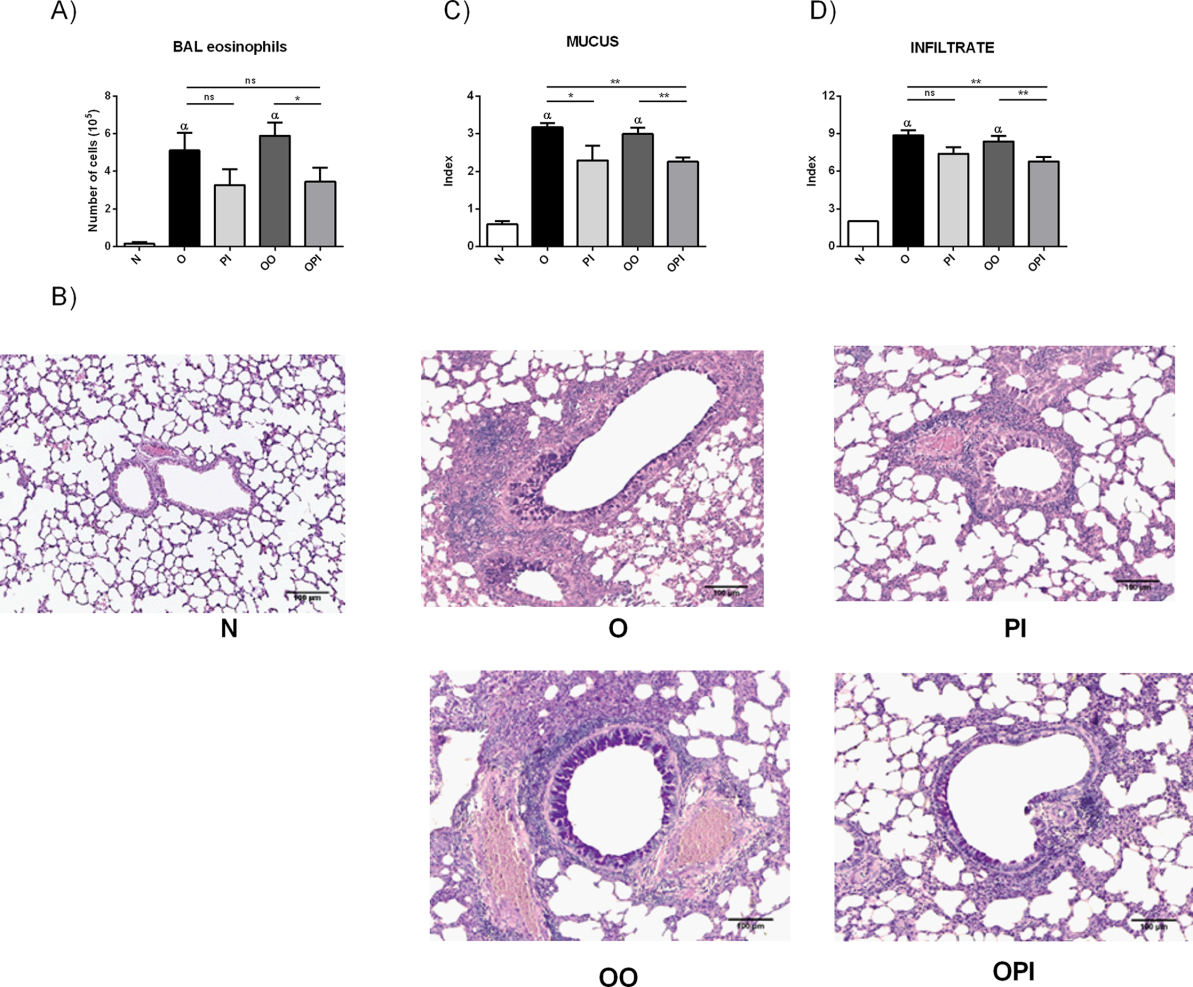
rTgPI-1 treatment ameliorates lung histopathology. (A) BAL eosinophil count was performed on cytocentrifuge slides stained with a modified Wright-Giemsa stain. (B) Sections for microscopy were stained with Haematoxylin and PAS. Original magnification 100X. (C) A histological goblet cell score was obtained in Periodic acid- Schiff (PAS)-stained lung sections by examining 20 consecutive airways at 400x magnification. (D) An index of pathologic changes in H&E slides was obtained by scoring the inflammatory infiltrate around the airways and vessels for greatest severity and overall extent. Results for each group are expressed as the mean ± SEM.*p <0.05 and **p <0.01, α *p*<0.01 vs N, ns: non-significant; ANOVA with Bonferroni's test *a posteriori*.

The effects of intranasal administration of rTgPI-1 were examined in a murine model of asthma. BALB/c mice were sensitized with two intraperitoneal inoculations of OVA/Alum and airway challenged with OVA. After 48 hours, allergic mice were intranasally treated for 3 days with rTgPI-1 alone (PI) or in combination with OVA (OPI). Positive controls included allergic mice intranasally treated with the allergen (OO) (positive control of OPI) or PBS (O) (positive control of PI), and the negative control included naive mice treated with rTgPI-1 (N). A week later, animals were re-challenged with the allergen and the asthma phenotype was evaluated (Fig 1). Consistent with an allergic inflammation, allergic mice intranasally exposed or not to the allergen (OO and O groups) presented a significant increase in BAL eosinophilia as compared with N mice (p<0.01) (Fig 3A). Animals treated with rTgPI-1 alone (PI group) presented a tendency to reduced numbers of this population. On the other hand, treatment with rTgPI-1 along with OVA (OPI group) resulted in a significant decrease in BAL eosinophils (Fig 3A).

The H&E and PAS lung-stained sections were analyzed to evaluate whether diminished BAL eosinophilia correlated with reduced lung pathology (Fig 3B). Both groups of sensitized and subsequent airway challenged mice (OO and O) showed typical pathologic features of pulmonary allergic inflammation compared with naive mice (N). These features include cell infiltration around airways and vessels, and mainly goblet cell metaplasia. Both groups treated with rTgPI-1 showed a decreased inflammatory infiltrate and goblet cell metaplasia (Fig 3B). However, the results of semiquantitative scoring of histology show that treatment with rTgPI-1 alone significantly decreased only mucus index while rTgPI-1 plus OVA diminished both parameters, mucus and infiltrate. Moreover, the improvement obtained with rTgPI-1 plus OVA was also observed when compared with asthmatic mice exposed to PBS (O control group), showing that this co-treatment was more effective than rTgPI-1 alone (Fig 3C and 3D).

### Effects of rTgPI-1 treatment on OVA-specific serum antibodies

**Fig 4:**
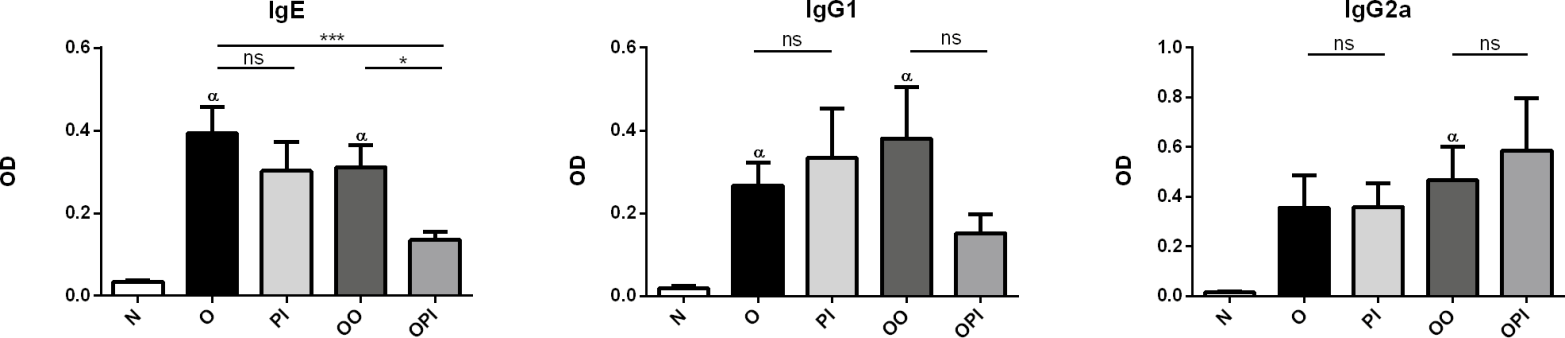
Effect of rTgP-1 treatment on allergen-specific humoral response. Serum levels of OVA-specific IgE (dilution, 1:40), IgG1 (dilution, 1:64x10^6^) and IgG2a (dilution, 1:16x10^3^) antibodies were quantified in all experimental groups. Results are expressed as the mean ± SEM. *p <0.05, α *p*<0.05 vs N, ns: non-significant; ANOVA with Bonferroni's test *a posteriori*.

Forty eight hours after the last OVA challenge, serum samples were obtained to analyze whether rTgPI-1 treatment could modulate OVA-specific immunoglobulin levels. As expected, IgE antibodies were increased in both groups of allergic mice (O and OO) compared to naive group. Intranasal rTgPI-1 administration concomitantly with allergen (OPI) significantly reduced OVA-specific IgE levels by nearly 56%. In addition, when compared to asthmatic mice exposed to PBS (O control group) this reduction reached 65% (Fig 4). Although not statistically significant, animals treated with rTgPI-1 alone showed a trend to diminished IgE antibodies. Serum concentration of neither IgG1 nor IgG2a isotypes showed significant changes (Fig 4).

We also considered important to evaluate whether as a consequence of the protocol treatment, a rTgPI-1 specific humoral response was induced. Interestingly, no specific antibodies against this protein were detected (S1 Fig). Moreover, intranasal immunization with rTgPI-1 with CpG-ODN in a vaccination protocol against *T*. *gondii* infection did not induce the production of rTgPI-1 specific antibodies ([32] and S1 Fig).

### rTgPI-1 treatment ameliorates OVA-specific airway resistance in allergic mice

**Fig 5:**
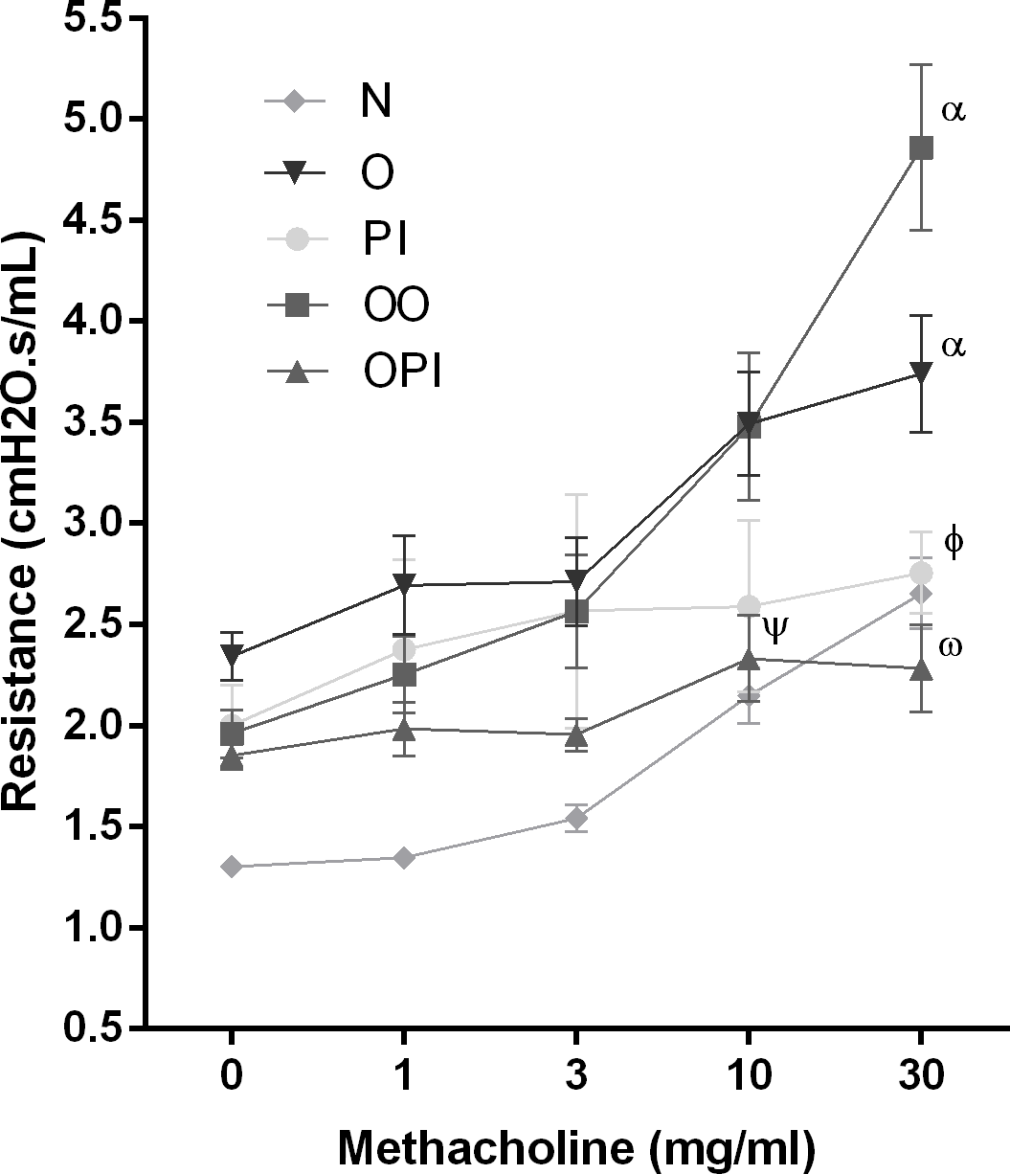
rTgPI-1 treatment attenuates airway hyperresponsiveness. Changes in pulmonary resistance in response to increasing doses of methacholine (0–30 mg/ml) were evaluated by invasive plethysmography. Results for each group are expressed as the mean ± SEM. ψ *p*<0.01 OPI vs OO and O, Ѡ *p*<0.001 OPI vs OO and O, φ *p*<0.05 PI vs O, α *p*<0.05 O and OO vs N; ANOVA with Bonferroni's test *a posteriori*.

The airway hyperresponsiveness (AHR) is a hallmark of bronchial asthma and determinant to make a positive diagnosis in patients. To study whether in addition to reducing lung inflammation, the rTgPI-1 treatments are able to improve lung function, 24 h after the last allergen challenge all groups of mice were subjected to methacholine stimulation testing. Compared to the naive group both O and OO groups showed increased airway resistance. Mice treated with rTgPI-1 either alone (PI) or co-administered with OVA (OPI) showed significantly reduced airway resistance compared to allergic mice (Fig 5). However and interestingly, the reduction was more remarkable with the rTgPI-1-OVA mixture than when mice were treated with rTgPI-1 without the allergen since a significant reduction of airway resistance was obtained at lower doses of methacholine (10 mg/ml) (Fig 5). Improvement in lung function was also analyzed by comparing the ratio between values obtained at the methacholine higher dose from the positive control group and its corresponding experimental group. This analysis showed an improvement of 53% (OPI) vs 26.3% (PI). Furthermore, even when compared to O group, co-treatment reduced airway resistance by 38%. These results not only indicate that intranasal administration of rTgPI-1 significantly ameliorated airway hyperresponsivenes in allergic mice, but also that co-administration of rTgPI-1 plus allergen resulted more effective than treatment with rTgPI-1 alone.

### Effects of rTgPI-1 treatment on cytokine production

**Fig 6:**
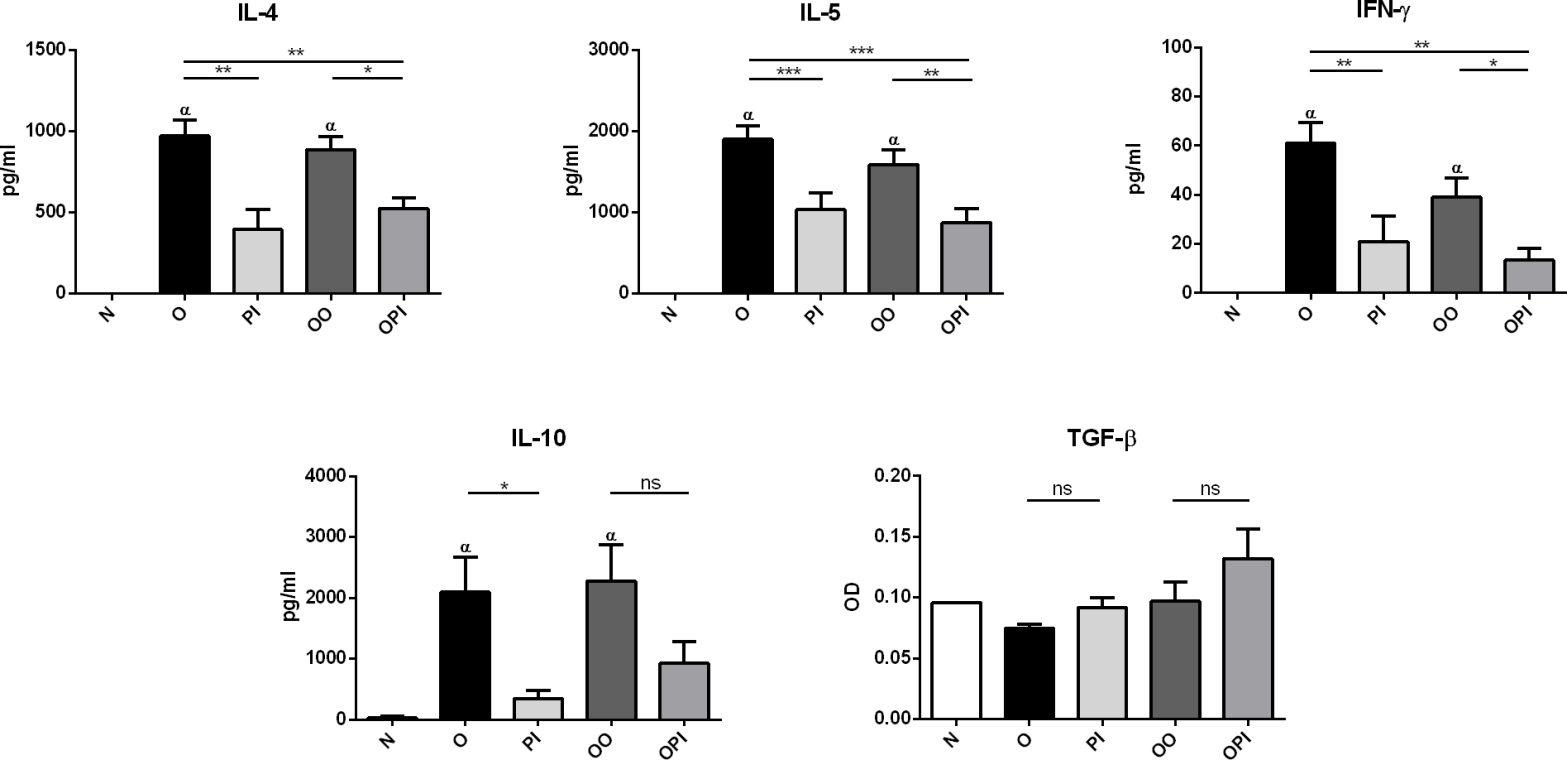
Local production of cytokines. Cytokine production by TLNC cells cultured *ex vivo* with OVA were analysed in all group of mice by ELISA. Results for each group are expressed as the mean ± SEM *p<0.05, **p<0.01, ***p<0.001, α p<0.001 vs N, ns: non-significant; ANOVA with Bonferroni’s test *a posteriori*.

We subsequently examined the effect of rTgPI-1 treatment on cytokine production by thoracic lymph node (TLN) cells *in vitro* stimulated with OVA. The levels of IL- 4, IL-5 and, to a lesser extent IFN-γ, were increased in sensitized and challenged mice intranasally exposed to PBS (O) or OVA (OO) compared to non-sensitized mice (N). Treatment with rTgPI-1 significantly decreased the levels of both Th2 (IL-4, IL-5) and Th1 (IFN-γ) type cytokines compared to the allergic groups (Fig 6). Also, while high levels of regulatory IL-10 were detected in supernatants from OVA-stimulated cells from allergic mice, a marked reduction was observed in both groups of animals treated with rTgPI-1. No significant differences were detected in allergen specific TGF-β production between groups (Fig 6).

### rTgPI-1 plus OVA treatment decreases allergen-induced T cell proliferation and expands FoxP3^+^Tregs

**Fig 7:**
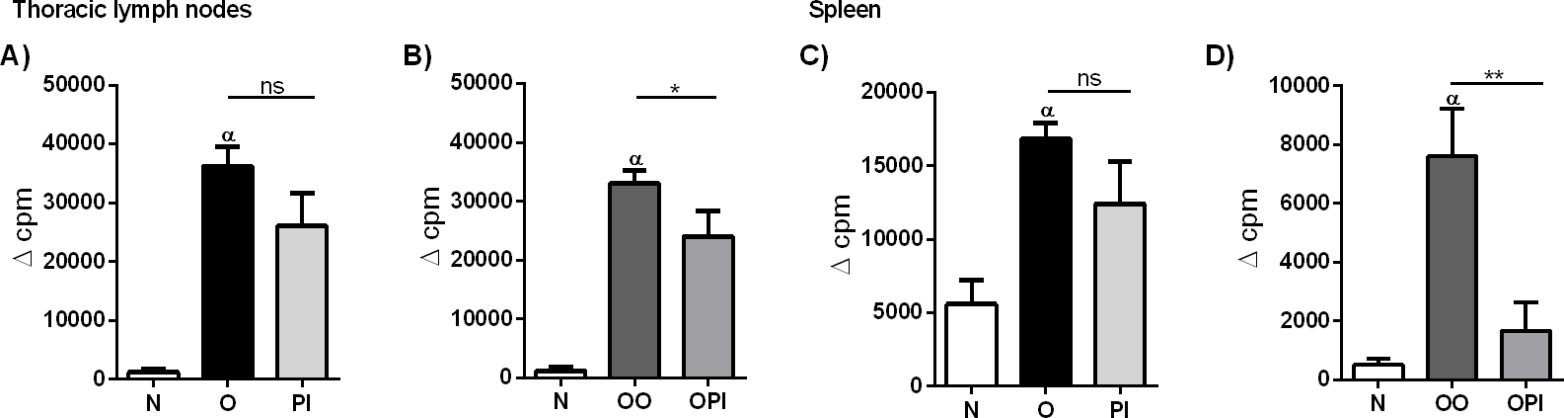
rTgPI-1 treatment suppresses cell proliferation. Proliferative responses of TLNC (A and B) or splenocytes (C and D) were determined by ^3^H-thymidine incorporation upon stimulation with OVA. Results for each group are expressed as the mean ± SEM. *p<0.05, **p<0.01, α *p*<0.001 vs N, ns: non-significant; ANOVA with Bonferroni's test *a posteriori*.

Successful immunotherapy significantly generates regulatory cells and reduces allergen-specific T cell proliferation, indicating the induction of peripheral tolerance [34–36]. Hence, we first evaluated allergen-specific TLN cell proliferation by ^3^H-thymidine incorporation during *ex vivo* culture in the presence of OVA. Co-administration of rTgPI-1 with OVA led to decreased proliferative responses (Fig 7B). In addition, splenocytes from the different experimental groups were *in-vitro* stimulated with OVA in order to evaluate rTgPI-1 modulation at systemic level. Interestingly, intranasal treatment with OVA+rTgPI-1 resulted in a dramatic decrease in allergen-specific T cell proliferation compared to allergic mice (Fig 7D).

**Fig 8:**
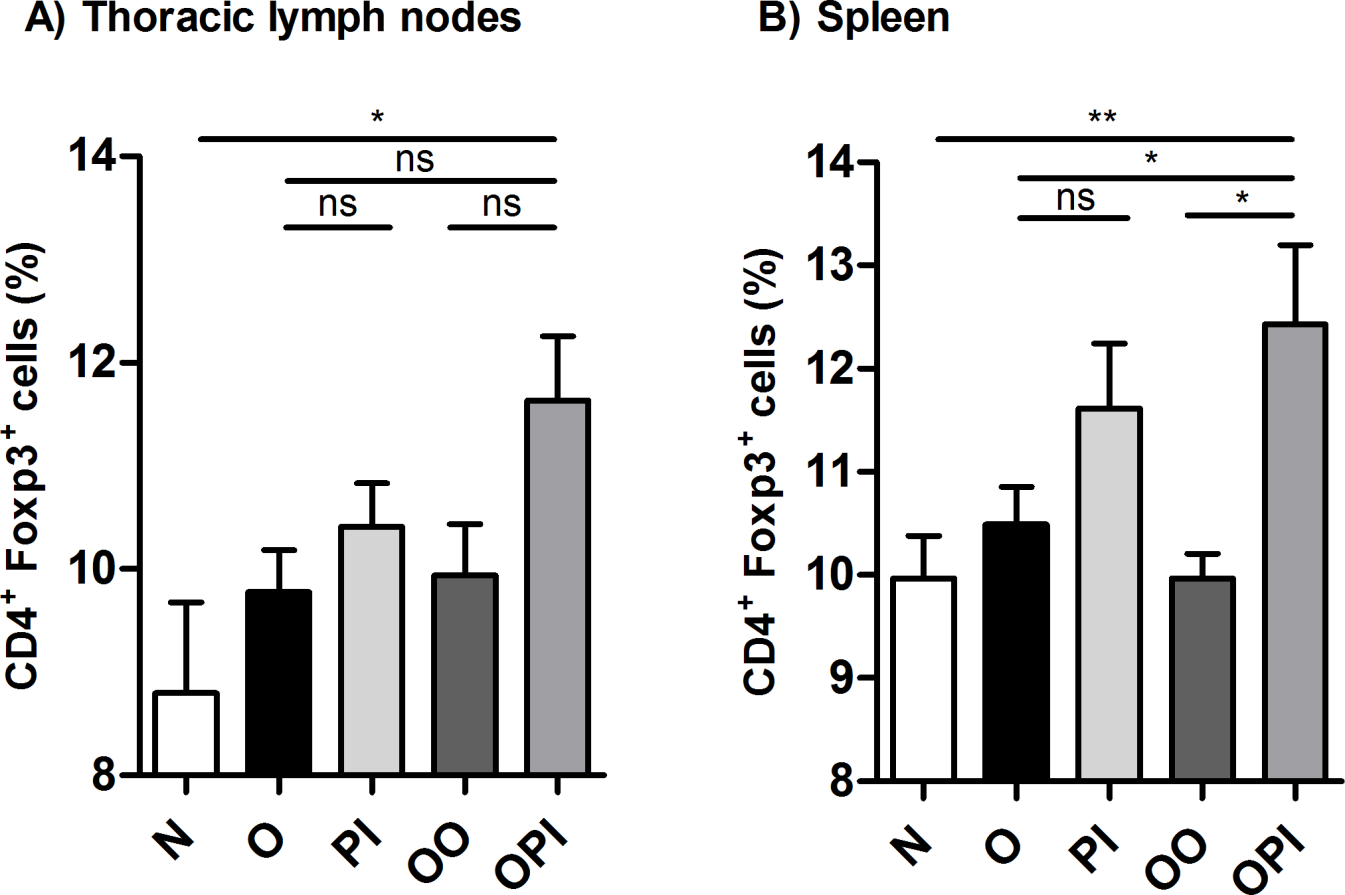
Co-administration of rTgPI-1 plus allergen enhances generation of Tregs both local and systemically. Tregs were identified by flow cytometry. The percentage of FoxP3^+^CD4^+^ among total CD4^+^ cells from TLNC (A) or splenocytes (B) was calculated. Results for each group are expressed as the mean % CD4^+^FoxP3^+^/CD4^+^ ± SEM. *p<0.05, **p<0.01, ns: non-significant; ANOVA with Bonferroni's test *a posteriori*.

To assess whether the diminished allergen specific proliferation observed in OPI treated mice correlated with increased CD4^+^FoxP3^+^cells, we evaluated the percentage of this population in thoracic lymph nodes and spleen from all experimental groups. An expansion of CD4^+^FoxP3^+^ cells in lymph nodes was detected only in animals that had been co-administered with the serine-protease inhibitor and OVA compared to naive mice (Fig 8A and S2 Fig). A significant increased percentage of Tregs in OPI group was also detected at systemic level not only compared to naïve (p< 0.01) but also to OO and O mice (p<0.05). Similar to lymph nodes, rTgPI-1 alone treatment didn’t result in a higher frequency of CD4^+^FoxP3^+^ cells (Fig 8B and S2 Fig).

## Discussion

The prevalence of IgE-mediated allergies is increasing worldwide. Factors including fewer microbial infections in association with the geographical spread of allergens as a consequence of global climate changes are potentially contributing to the rising prevalence of allergic disorders. Steroids, the major therapeutic agents for these disorders, are not an ideal treatment because of the appearance of adverse side effects associated with long-term use [2–4]. Therefore, new strategies for the treatment of IgE-mediated disorders have become an important area of investigation. Allergen-specific immunotherapy has emerged as an alternative treatment. Still, at present, this kind of therapy presents several concerns related to the type of vaccine and adjuvant used, route of inoculation, side effects and limited effectiveness.

We have previously demonstrated that both acute and chronic *T*. *gondii* infection strongly diminish the development of an allergic airway inflammation [33]. The mechanisms behind this phenomenon included the deviation to a Th1 phenotype plus the induction of regulatory cells [37,38] that might result from the activity of many different immunomodulatory proteins.

TgPI-1 is a *Toxoplasma gondii* protein able to inhibit many serine proteases including trypsin, chymotrypsin, neutrophil elastase [12] and subtisilin [13]. It has been shown that many serine protease inhibitors could modulate the immune response. The endogenous serine protease inhibitor produced by human epithelial cells (SLPI), inhibits inflammatory responses not only by blocking the proteolytic activity of serine proteases released by leukocytes, but also by down modulating LPS induced pro-inflammatory cytokines [14,39]. In addition, SLPI directly affects monocytes by modulating their cytokine secretion pattern, which in turn inhibits the proliferation of CD4^+^ T cells and Th1 cytokine secretion [40]. Administration of human serum protease inhibitor α1-antitrypsin (hAAT) reduced pro-inflammatory cytokine production via direct modulation of dendritic cell responses [41], enhanced IL-10 secretion and expanded Tregs [16,17,42] in many *in vivo* experimental models. More recently, it has been shown that B cells are cellular targets of hAAT. Moreover, hAAT-induced Treg cell expansion appears to be B-cell-dependent [18]. These observations showing the immunomodulatory properties of serine-proteases inhibitors, suggest the potential of these molecules as adjuvant candidates for immunotherapy.

It is known that many proteases play key roles in asthma pathophysiology [19–21]. Trypsin [26], mast cell tryptase [20] and neutrophil elastase [25] are known to be the endogenous stimuli for protease-activated receptor 2 (PAR-2). This receptor is expressed by a variety of cells including airway epithelial cells, fibroblasts, myocytes, sensory neurons, and bronchial and vascular smooth muscle [43], and its activation has been shown to play a role in the development of AHR and airway inflammation [44]. It was also shown that in OVA-induced experimental asthma models, OVA challenges would activate an airway endogenous protease required for allergic lung disease development [19].

All this background let us hypothesized that TgPI-1 might reduce allergic airway inflammation not only by inhibiting serine proteases involved in asthma but also by acting as a tolerogenic molecule.

To our knowledge, few previous studies have evaluated serine-protease inhibitors as experimental asthma modulators [27–30,45]. In most of them the inhibitor was administered alone during allergen sensitization or during the primary challenge [28–30]. Herein we explored the effect of a recombinant form of TgPI-1 by using a protocol in which the inhibitor was administered alone or along with the antigen, to mice with already established asthma. Additionally, a week later animals were given a secondary challenge with the allergen. Moreover, herein mice were treated intranasally instead of intraperitoneally as previously performed by Koga *et al*. [45] and Chen *et al*. [46]. We understand that our therapeutic model more closely resembles the human situation.

Both rTgPI-1 treatments, alone or co-administered with the allergen, reduced asthma hallmarks: allergic lung inflammation and more importantly airway hyperresponsiveness. Increases in the number of eosinophils in BAL, peribronchial cell infiltration and goblet cell metaplasia were all inhibited. However reduction in airway resistance was more remarkable with the rTgPI-1-OVA mixture than when mice were treated with rTgPI-1 without the allergen since a significant reduction of airway resistance was obtained at lower doses of methacholine (10 mg/ml). In addition, by comparing the ratio between pulmonary resistance obtained at the methacholine higher dose from each treated group and its corresponding positive control group, an improvement of 56% in OPI vs 26% in PI was observed. Furthermore, even when compared to O group, co-treatment reduced airway resistance by 38%. The difference between mice treated with rTgPI-1 and rTgPI-1 plus allergen was also remarkable for allergen specific IgE production. Only mice treated with OVA plus rTgPI-1 showed a significant reduction in OVA specific IgE compared to both asthmatic groups. These results let us to conclude that treatment with rTgPI-1 concomitantly with the allergen was more effective.

On the other hand, the fact that i.n. OVA administration along with rTgPI-1 diminished all asthma hallmarks while exposure to OVA alone didn’t modify the asthma outcome may be explained solely by an anti-inflammatory ability of the protease inhibitor. However, since OVA+rTgPI-1 treatment resulted more effective than rTgPI-1 alone, our results suggest that rTgPI-1 also has adjuvant capacity. Hence, the more effectiveness observed in the co-treatment might be explained by the addition of these two properties.

To address the underlying mechanisms whereby rTgPI-1 modulates allergen-induced airway inflammation and AHR, TLN cytokine levels were measured. We found that not only the Th2 cytokine levels were profoundly inhibited by rTgPI-1 or rTgPI-1+OVA treatment, but also the Th1 cytokine IFN-γ, arguing that allergy inhibition was not simply achieved by immune deviation toward a Th1 profile. As we have previously mentioned, effective immunotherapy should result in the induction of allergen specific peripheral tolerance showed as decreased allergen T cell proliferation [34,35] and increased Tregs [36]. In this regard and interestingly, our intranasal treatment protocol with rTgPI-1 plus OVA significantly diminished OVA-specific proliferation not only locally in TLNC but also systemically. Regulatory IL-10 cytokine down-modulates both type 1 and type 2 cytokines and induces anergy of effector T cells [47]. Effective treatment with AEBSF [27,48] or nafamostat mesilate [46] on experimental asthma induced the production of IL-10. More recently, Kim *et al*. showed that, in addition to increased levels of IL-10, treatment of allergic mice with AEBSF induce CD4^+^CD25^+^FoxP3^+^ T cells [48]. Our results do not support a role for this cytokine in this protocol since IL-10 was also diminished in both treated groups. On the other hand, although a trend to increased TGF-β levels was observed when rTgPI-1 was administered along with OVA, no significant differences were registered. Still, the reduced proliferative response of TLN cells and splenocytes upon OVA stimulation, along with the diminished Th1 and Th2 cytokine secretion suggested the presence of regulatory cells in the treated groups. In effect, when OVA was administered with rTgPI-1, significant increased frequency of CD4^+^FoxP3^+^ T cells were detected both in thoracic lymph nodes and spleen. On the other hand, treatment with rTgPI-1 alone didn’t result in higher levels of CD4^+^FoxP3^+^ cells nor in a significant reduced OVA-specific T-cell proliferation. These results might explain the better outcome of co-administration of rTgPI-1 with the allergen.

The fact that targeting proteolytic activity by synthetic inhibitors such as AEBSF [27,48] or nafamostat mesilate [46] reduces allergic airway inflammation in a mouse model suggests that inhibition of at least some serine proteases should be sufficient for the decrease of the severity of the disease. However, the effect of rTgPI-1 on allergic inflammation could be given not only by blocking the activity of proteases but also by still unknown immunomodulatory properties independent of its anti-protease activity. Thus, rTgPI-1 may act by targeting other molecules beyond serine proteases. In this regard, Jonigk *et al* showed evidence that the anti-inflammatory and immunomodulatory properties of AAT can be independent of elastase inhibition [49].

Hence, the anti-allergic effect of rTgPI-1 could be explained by several pathways: by inhibiting serine proteases known to be involved in asthma and by inducing Tregs which suppresses allergic responses via their ability to inhibit several cell types involved.

## Conclusions

Overall, we found that intranasal administration of rTgPI-1 to allergic mice exerts a therapeutic effect not only by ameliorating pulmonary inflammation but also by improving lung function. Although further studies should be undertaken to completely elucidate the detailed mechanisms by which this *T*. *gondii* serine protease inhibitor treatment improves asthma phenotype, rTgPI-1 appears as a promising candidate for intervention in patients with asthma. Furthermore, treatment with rTgPI-1 seems to be more effective when administered along with the allergen suggesting that this inhibitor may have adjuvant properties. These results open the window to future studies to evaluate the adjuvant ability of rTgPI-1 in allergen specific immunotherapy experimental models.

## Supporting information

S1 Fig rTgPI-1 treatment doesn’t induce the production of specific antibodies. Forty eight hours after the final challenge, the total circulating volume of blood was taken, serum was collected, and TgPI-1-specific IgG (dilution, 1:250), was quantified in naive (N), allergic mice treated with rTgPI-1 (PI), and allergic mice treated with OVA plus rTgPI-1 (OPI). Serum from mice intranasally immunized with rTgPI-1 with CpG (TgPI+CpG) or intramuscularly immunized with rTgPI-1 with Alum (TgPI+Alum) were used as controls. Results for each group are expressed as the mean ± SEM. ***, *p* <0.001, ns: non-significant; ANOVA with Bonferroni's test *a posteriori*.
(TIF)

S2 Fig CD4^+^FoxP3^+^ T cells in draining lymph nodes and spleen. Flow cytometry analysis of CD4^+^FoxP3^+^ T cells from N, O, PI, OO and OPI mice. Representative dot plots from each group are shown.
(TIF)
