# Supplementary figures and images for: Toxoplasma gondii serine-protease inhibitor-1: A new adjuvant candidate for asthma therapy
Source: PLoS One. 2017 Oct 26;12(10):e0187002. doi: 10.1371/journal.pone.0187002 (PMC5658115; doi:10.1371/journal.pone.0187002)

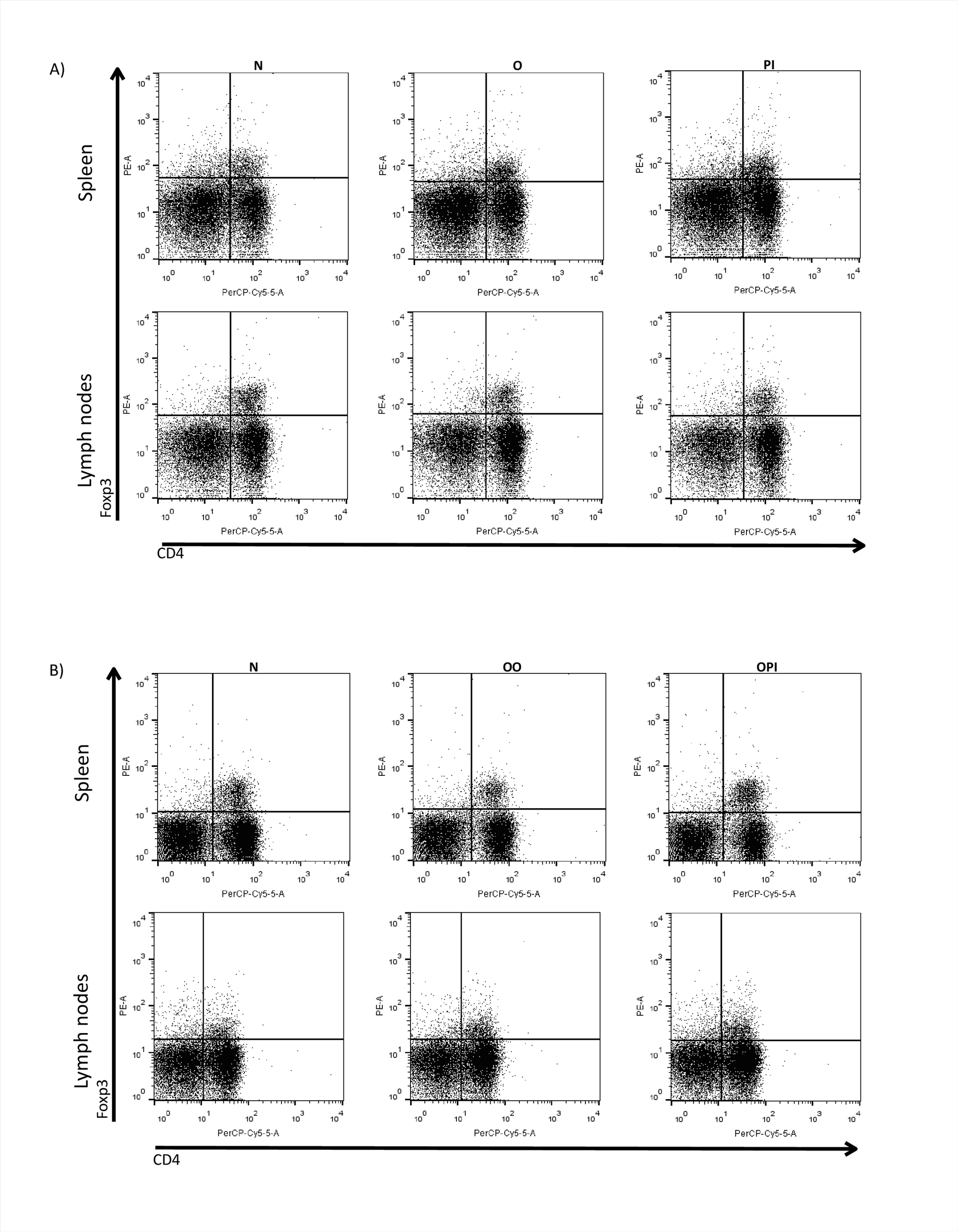

Supplement: S2 Fig — Flow cytometry analysis of CD4+FoxP3+ T cells from N, O, PI, OO and OPI mice. Representative dot plots from each group are shown. (TIF) [file pone.0187002.s002.tif]
